# Singing for Lung Health—a systematic review of the literature and consensus statement

**DOI:** 10.1038/npjpcrm.2016.80

**Published:** 2016-12-01

**Authors:** Adam Lewis, Phoene Cave, Myra Stern, Lindsay Welch, Karen Taylor, Juliet Russell, Anne-Marie Doyle, Anne-Marie Russell, Heather McKee, Stephen Clift, Julia Bott, Nicholas S Hopkinson

**Affiliations:** 1 NIHR Respiratory Biomedical Research Unit at Royal Brompton and Harefield NHS Foundation Trust and Imperial College London, London, UK; 2 Respiratory Medicine, Whittington Health, London, UK; 3 Southampton Integrated COPD Service, Solent NHS Trust, Southampton, UK; 4 British Lung Foundation, London, UK; 5 Sidney De Haan Research Centre for Arts and Health, Canterbury Christ Church University, Canterbury, UK

## Abstract

There is growing interest in Singing for Lung Health (SLH), an approach where patients with respiratory disease take part in singing groups, intended to improve their condition. A consensus group was convened in early 2016 to address issues including: the specific features that make SLH distinct from other forms of participation in singing; the existing evidence base via a systematic review; gaps in the evidence base including the need to define value-based outcome measures for sustainable commissioning of SLH; defining the measures needed to evaluate both individuals' responses to SLH and the quality of singing programmes. and core training, expertise and competencies required by singing group leaders to deliver high-quality programmes. A systematic review to establish the extent of the evidence base for SLH was undertaken. Electronic databases, including Pubmed, OVID Medline and Embase, Web of Science, Cochrane central register of controlled trials and PEDro, were used. Six studies were included in the final review. Quantitative data suggest that singing has the potential to improve health-related quality of life, particularly related to physical health, and levels of anxiety without causing significant side effects. There is a significant risk of bias in many of the existing studies with small numbers of subjects overall. Little comparison can be made between studies owing to their heterogeneity in design. Qualitative data indicate that singing is an enjoyable experience for patients, who consistently report that it helps them to cope with their condition better. Larger and longer-term trials are needed.

## Background

Many people with chronic respiratory conditions experience breathlessness, which is both limiting and distressing, despite the availability of therapies, including smoking cessation, pulmonary rehabilitation (PR) and medication.^1^ Singing for Lung Health (SLH) also referred to as ‘Singing for Breathing’ has emerged as a novel approach to address this.^2–5^ It involves the delivery of classes led by a singing teacher with relevant skills and experience. The learning of techniques around breathing control and posture that are necessary to sing effectively is combined with a group activity that is perceived as fun and sociable. The goal of the groups is to become able to produce song, an artistic objective, but through this process individuals acquire skills to help them to cope with their lung condition, a health-improvement objective. This paper includes a systematic review of evidence of clinical benefit for singing in respiratory disease followed by a consensus group statement on SLH.

## Systematic literature review—singing as a therapeutic intervention for patients with respiratory conditions

A systematic review was required to allow the consensus group to judge how firmly recommendations around SLH as a clinical intervention can be made. To the authors’ knowledge, no systematic review exists investigating singing specifically related to groups of people with respiratory conditions. The objective of this systematic review was to address the question ‘Does singing improve the health of people with respiratory disease?’ by collating published evidence on singing as a therapy for chronic respiratory diseases, including chronic obstructive pulmonary disease (COPD), bronchiectasis, interstitial lung disease, obstructive sleep apnoea or asthma. Types of studies included randomised controlled trials (RCTs), controlled trials and cohort studies that enrolled adults with an established diagnosis of a chronic respiratory disease. The review was registered on the PROSPERO database: CRD42016037705.

Participation in a singing group with the intention of improving the person’s respiratory condition was compared with standard care or a control treatment. Outcomes included measures of lung function, health status, quality of life and functional exercise capacity. Electronic databases (Pubmed, OVID Medline and Embase, Web of Science, Cochrane central register of controlled trials, PEDro) were used to search for the terms ‘singing’ AND (‘respiratory disease’ OR ‘COPD’ OR ‘asthma’ OR ‘Bronchiectasis’, OR ‘interstitial lung disease’ OR ‘obstructive sleep apnoea’).

The search was run on 14 April 2016. Limits to the search were added, including no related terms, only adult population, a time period of 1980 to current and results obtained with a five-star rating (results including both ‘singing’ and ‘respiratory disease’ or specific pathology name). An example of a database search can be found in Supplementary Appendix SA. Six hundred and nine results were returned from the above databases. Two further articles were obtained after reviewing article references and contacting authors. Four hundred and nineteen articles remained following de-duplication across databases. Titles, abstracts and articles were reviewed by AL. After AL screened the titles and de-duplicated the results, 22 titles appeared to be of further interest. Sixteen of these studies were excluded. Studies were excluded if singing was delivered as part of a PR programme because it would be difficult to separate the effects of either group intervention. Others were excluded if they were literature reviews or conference proceedings only. Details of excluded studies are given in Supplementary Appendix SB. Primary outcomes included those measuring health status (36-item short form survey (SF-36), COPD Assessment Test, St George’s Respiratory Questionnaire, Clinical COPD Questionnaire, Chronic Respiratory Disease Questionnaire, King’s Brief Interstitial Lung Disease Questionnaire), exercise capacity (Six Minute Walk Test, Incremental Shuttle Walk Test, Endurance Shuttle Walk Test) and lung function (forced expiratory volume in 1 s (FEV_1_), forced vital capacity (FVC), the FEV_1_ over FVC ratio (FEV_1_/FVC), inspiratory capacity, maximal inspiratory pressure, maximal expiratory pressure). Secondary outcomes included patient experience. Qualitative data were identified with the inclusion of verbal or written comments. An assumption was made that qualitative data collection and analysis was completed following appropriate qualitative research methodology.

**Figure 1:**
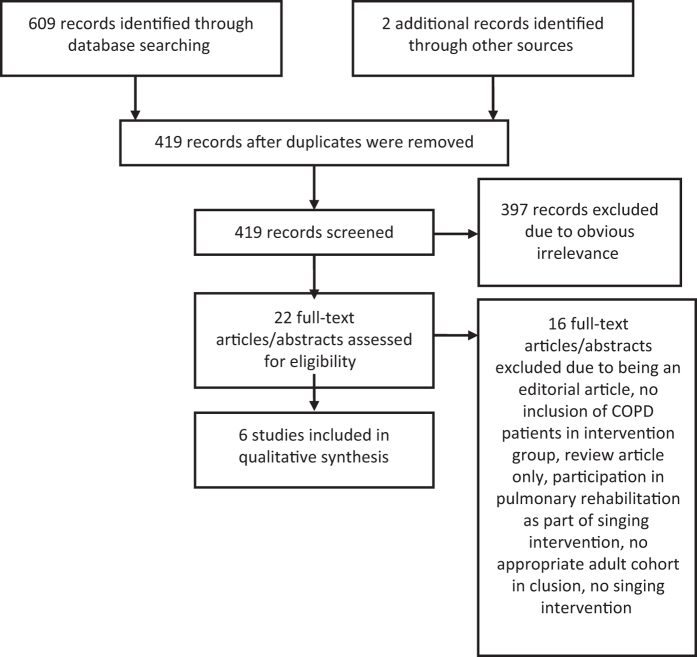
PRISMA flow diagram of results.

Data extraction from studies included in the final review was carried out independently by AL on a paper-by-paper basis and put into a table of results found in Supplementary Appendix SC. Because of the heterogeneity in outcome measures used across studies, it was decided to report them narratively. Contact was made with Rebecca Engen, author of an excluded study, to clarify subjects' participation in PR during the singing intervention. The variables for which the data were sought are outlined in Table 1 including characteristics of trial participants, type and description of intervention and control and type of outcome measures used. Six studies were included in the review (Table 1); four RCTs^2,3,6,7^ and two cohort studies.^4,8,9^ Three of these studies were performed in the United Kingdom, one in Brazil, one in Canada and one in Australia. A flow diagram of the results is included in Figure 1.

Assessment of risk of bias was performed at study level using the Cochrane Collaborative Tool (Table 2). No meta-analysis or combining results was performed, and no summary measures were used because of a lack of repeated outcome measures across all studies.

### Randomised controlled trials

Lord *et al.*^2^ performed a trial in COPD patients comparing twice weekly 1 h singing classes (provided by a single teacher) for 6 weeks against usual care. Of the 36 participants enrolled, paired data were available from 15 singers and 13 controls who completed the study. Data were analysed on an intention-to-treat basis. Singing was associated with an improvement in the SF-36 physical component score (+7.5 (14.6) vs. −3.8 (8.4) *P*=0.02) and the Hospital Anxiety and Depression anxiety score (−1.1 (2.7) vs. +0.8 (1.7) *P*=0.03). Although these improved results from SLH are statistically significant, caution is required in interpreting the clinical significance of the findings owing to the low patient numbers and the s.d. being greater than the means. There were no differences in walking distance (+26 (52.6)+11.3 (83.0) *P*=0.58). Interestingly, there was a significant increase in breath hold time after intervention in the control group (5.3 s (5.7)) compared with the singers (−0.3 s (6.9)) *P*=0.029. The authors argue that this apparently paradoxical result may have been due to those in the singing training group learning to take a more measured inspiration. The breath hold test is anyway more suitable to evaluate interventions for people with hyperventilation than COPD.

A second RCT from Lord *et al.*,^3^ again in COPD patients, compared the effect of a longer duration of singing classes, this time for 8 weeks, with participation in a film study group. The primary outcome was change in the SF-36. Follow-up data were available from 13 participants in the singing arm and 11 participants in the film group arm, with attrition of 5 participants from the singing arm and 3 from the film arm. The mental component score of the SF-36 improved to a similar extent in both groups; 9.3 (25.3) vs. 4.3 (9.0) in the singers versus film group (*P*=0.41). However, there was a significantly greater improvement in the SF-36 physical component score in the singing arm (12.9 (19.0) vs. −2.5 (11.9) *P*=0.02). There were no statistically significant differences in exercise capacity or physical activity level between groups. Interestingly, breath hold time again fell in the singing group compared with controls though this difference was not significant (−1.64 (4.1) vs. 2.39 s (7.8) *P*=0.14)). The greater improvement in SF-36 Physical Component Summary scores from this study compared with the groups previous study may be due to the longer intervention period. This may suggest that singing groups for respiratory conditions need to run for at least 8 weeks and physical health-related quality of life may continue to improve with longer duration interventions. However, the confidence intervals of SF-36 Physical Component Summary results in both studies are wide and therefore further research is required before reaching this conclusion.

In both these studies, all participants received a single 30-min session of breathing control instruction from a senior physiotherapist as part of the intervention. This education may help define SLH compared with singing for well-being groups but may be a confounding factor when interpreting between-group differences.

Bonilha *et al.*^6^ performed an RCT in COPD patients comparing 1 h once a week group singing lessons to once a week handicraft sessions for 24 weeks. The randomisation process is not reported. Participant attrition from the initial 43 participants randomised left 15 participants in each group at the end of the study. The authors based their sample size of 15 people in each group on a previous study, which detected a between-group difference in maximal respiratory pressures after training in COPD patients. Singing was well tolerated. Quality of life improvements did not differ between groups, but patients in the singing group had increased maximal expiratory pressure 1 week after the final class, whereas this had fallen in the control arm (+3.0 (17.2) vs. −11.3 cm H_2_O *P*=0.05). The authors report a transient increase in inspiratory capacity measured 2 min after finishing singing in the last session (0.14 (0.25) vs. −0.08 (0.18) litre *P*=0.01). Singing also improved oxygen saturations during singing compared with the control group (1.6 (1.8)% vs. 0 (1.2)% *P*=0.01).

Gick and Daugherty^7^ performed an RCT in a cohort of patients with a self-reported diagnosis of asthma. Sixty participants took part for 4 weeks in weekly singing classes, singing classes with diaphragmatic breathing instruction or just diaphragmatic breathing instruction classes. They were also encouraged to practice at home and keep a log of this. The choice of repertoire for the songs was not specifically targeted to help with asthma management. Popular songs were chosen by graduates of the participating university. Partial block randomisation was used with a block size of three. It is not stated how the randomisation was performed. Attrition in each group ranged from 25% to 16% and did not differ significantly. However, more men (40%) than women (13.3%) dropped out. Following the training, all groups showed significant improvements in measures, including peak expiratory flow rate, quality of life, mood and breathlessness, but the authors report a lack of outcome differences between the three arms. There was no further analysis of direct comparison of singing and breathing intervention with the breathing instruction alone intervention and no usual care control group.

### Cohort studies

Eley *et al.*^8,9^ report two cohort studies including Aboriginal Australian children and adults with a diagnosis of asthma. Female participants had singing lessons, whereas males participated in didgeridoo classes. It was unclear in the study^9^ which patients in the second community group were adults as the age range for females spanned 7–35 years. There were no significant changes in spirometry values, but peak expiratory flow rate improved. Daily peak expiratory flow rate values were highly variable with no significant change across the course of the study. Moreover, the retention of female adult asthmatics was poor^10^ and final outcome data were only available from three participants.

Morrison *et al.*^4^ performed a feasibility study to investigate the potential of establishing a community-based singing programme for COPD patients.^4^ Patients were recruited by mailing COPD registered patients on GP practice databases in East Kent, by newspaper advertisements and by direct contact with local patient support groups. One hundred and six patients took part in weekly 90-min singing sessions over 36 weeks. The St George’s Respiratory Questionnaire total score improved by 3.3 (6.14–0.45) points at the end compared with baseline, but there were no significant changes in Medical Research Council Dyspnoea Score, SF12 or EQ-5D.

### Qualitative studies and data within RCTs and cohort studies

In addition to outcomes from the RCT, Lord *et al.*^2^ also reports on an additional 150 participants in open singing workshops, run in the same hospital, who completed a questionnaire about the experience. In all, 96% rated the workshops as ‘very enjoyable’ and 98% thought the workshop had taught them something about breathing in a different way. Also, 81% of attendees felt a ‘marked physical difference’ after the workshop.

Structured interviews with eight COPD patients were completed after completion of the initial trial by Lord *et al.*^2^ Participants described a positive impact from the singing groups relating to both physical and general well-being. Singing contributed to an increased sense of breath control. Positive impacts on patient’s posture, walking, ability to control breathlessness during an exacerbation and housework were reported as a result of doing singing in the group. The benefits of enjoyment, improved mood and social contact were also reported.

Participants in the second RCT by Lord *et al.*^3^ described benefits from singing in terms of improvements in knowledge of breathing, improved breathing control and improved ability to do housework. Others, who had multiple health conditions, reported that it was difficult to ascertain any physical benefits from singing. Patients who had participated in the film group reported no physical benefits from this, although they had enjoyed it. In Eley *et al.*,^8,9^ only one quote could be attributed to an adult female: ‘it got me out of the house and mixing with other people’. There are insufficient other qualitative data to draw any conclusions from adult females in the study. Participants’ perceptions are reported in detail in Skingley *et al.*,^5^ with singers describing mental and physical benefits, including learning how to breathe properly, improved posture as well as social benefits, enjoyment, improved well-being, general physical health and a desire for the groups to continue.

## Discussion

There is a significant risk of multiple forms of bias across the studies with heterogeneous interventions and a limited number of participants. Performance bias was the biggest risk of bias in these studies as blinding participants is not possible. Some studies lacked information on randomisation procedure and so there was evidence of selection bias. Other studies included in the review were not controlled and therefore inherently carry a risk of bias. Moreover, attrition in the form of participant drop out was evident across studies. More information is required in the reporting of the singing exercises and other stages performed within studies to ascertain a potential therapeutic cause of this improved lung function measurements. Currently, it is not clear whether, when and how active abdominal contraction was encouraged during expirations or whether increased pressures through the hands on the upper abdomen were used as in active assistive mechanism (e.g., a manually assistive cough technique) or passive restrictive mechanism (e.g., a diaphragmatic breathing technique) in the study by Bonilha *et al.*,^6^ for example. Nevertheless, repeated reporting of improvements in different outcomes across studies suggests that SLH improves health status in COPD patients in particular. No significant adverse events or side effects have been reported. Larger and longer-term studies are needed to quantify and understand its effects and impact, but participation in SLH has the potential to improve management of breathlessness and increase social participation.

There is limited evidence from controlled trials that, to date, have been small scale, involving a total of 112 patients (43 completing a singing arm). Findings of note from controlled trials are an improvement in the SF-36 Physical Component Score, reduced breath hold time, an acute, transient increase in inspiratory capacity and improved maximal expiratory pressure.

There are further limitations to existing studies. No long-term follow-up after intervention is part of the method in any study, with significant attrition across studies, limiting the conclusions from the quantitative data in studies published. Singing interventions differ across studies and therefore make it difficult to ascertain the overall effect of respiratory therapy across the studies and no meta-analysis could be performed as a result. Results from outcome measures are not fully reported across studies and power calculations for sample sizes are not consistently provided. The singing interventions across studies are heterogeneous and lack detail about the nature of how singing was delivered as a therapeutic intervention to improve respiratory disease management or more specifically breathlessness. Future studies with larger sample sizes are required to confirm these findings. There are no trials available to ascertain the clinical importance of singing for adults who have bronchiectasis, interstitial lung disease or obstructive sleep apnoea. Furthermore, no data were available across studies on patient health-care utilization, such as exacerbation rates and admissions. These outcomes may be important to investigate in the future in order for commissioners to better judge the value of singing as an intervention compared with other therapies. Only scientific databases were used in the literature search and no performance or arts databases were used. Publication bias may be present in this review as the authors did not review unpublished studies or reports of results.

This systematic review demonstrates that there is considerable qualitative data to support participation in singing groups as a safe and potentially valuable strategy for people with COPD.

## Consensus statement on SLH

### Introduction

Most cultures incorporate public singing as a social activity and it is part of most people’s experience, certainly in childhood. Adult participation rates are lower and inversely associated with level of deprivation.^11^ Healthy people who take part in singing report benefits in terms of both social interaction and well-being.^12^ Observational studies have found that singing is generally experienced as enjoyable and helpful by respiratory patients.^2,10^ This is supported by the findings of small RCTs,^2,3,6,7^ suggesting that it can lead to improvements in health status. The majority of participants so far have had COPD or asthma. This has resulted in considerable interest, with numerous singing groups for people with lung disease now established across the United Kingdom. At present, participation in many of the groups is supported by The British Lung Foundation or through other charitable funding or by participants themselves rather than by commissioners of health or social care. A handful of groups have received some public health funding.

Given this rapidly changing landscape, a consensus group, involving the British Lung Foundation, health professionals (physiotherapy, nursing, health psychology, music therapy, respiratory physician) and arts practitioners with an interest in this area, was convened at Royal Brompton Hospital. The purpose of this group was to review the available evidence, identify outstanding issues around the role and delivery of SLH and consider solutions (see Table 3).

## Defining ‘Singing for Lung Health’

Singing is a complex activity, involving not only postural and breathing support but also phonation at the vocal fold level and resonation involving the movement of the tongue, soft palate and larynx. In addition, it requires adjustment of pitch and negotiating both range and volume while learning melodies ‘by ear’. SLH can be distinguished from participation in more generic singing activities by its focus on improving breath control and posture in relation to respiratory disease, using songs as tools for this purpose. These take precedence over the quality of singing produced and preparation for public performance, although efforts to improve the quality of singing provide an important impetus for ongoing participation. SLH groups include specific components around posture and breath control tailored to lung disease, in particular airflow obstruction. Cough is a common feature of respiratory disease and may limit participation in a conventional choir, whereas SLH groups represent a more tolerant environment.

A typical 60-min class would include physical warm-ups, breathing exercises, vocal warm ups, songs and a cool down as outlined in Table 4.

## By what mechanisms can SLH be beneficial?

The benefits of singing can be considered in three broad categories—physical, psychological and social—though these necessarily overlap and interact. Although breathing patterns are to an extent determined, or at least limited, by pathophysiological processes such as airflow obstruction and abnormal lung compliance, breathing pattern can be modified by conscious attention or training to influence use of inspiratory and expiratory muscles.^13^ For example, skills acquired during singing may help to avoid the rapid ‘breath-stacking’ pattern of breathing in COPD, where dynamic hyperinflation reduces inspiratory reserve volume and worsens breathlessness. Inspiratory capacity manoeuvres^6^ and chest wall kinematics confirm that singing lowers end-expiratory lung volumes in COPD.^14^

There is an established repertoire of physiotherapy techniques for helping to manage breathlessness in patients with COPD, including postural modification, breathing control, pursed lips breathing and ‘blow as you go’.^15^ These improve respiratory muscle and/or lung and airway mechanics, prolonging expiratory time, allowing a reduction in operating lung volumes. Controlling expiration through singing techniques may have analogous effects, being useful when managing and recovering from episodes of extreme breathlessness. Singing may also enhance sputum clearance as both dynamic lung volume and airflow are increased, features present in conventional physiotherapy techniques.^15^

A growing body of research demonstrates that COPD is affected by a range of behavioural and psychological pathways and is associated with high levels of psychological distress for example, anxiety and depression, which is often undetected and negatively impacts on health. Through complex pathways and interactions, psychological factors have been demonstrated to impact on: neurobiological perception of breathlessness and reduced dyspnoea thresholds^16^; reduced self-management behaviours, for example, medication/exercise/smoking adherence and management of exacerbations; progressive avoidance of activities, social withdrawal and social isolation; reduced quality of life; and overall worsening course of disease.^17,18^ SLH can positively influence a number of these pathways and thereby potentially contribute to improvements in physical and psychological health, social well-being and overall quality of life.

In particular, patients with respiratory disease usually experience their breathing in a negative way, as something which limits them. Singing, by allowing conscious attention to breathing in a context associated with the positive achievement of song, may help to reduce this negative association. Singing is an activity that a respiratory patient can do well, receiving well-earned praise and thus boosting self-esteem. Participation in a group activity with other people with a lung disease facilitates modelling of effective self-management behaviours, the exchange of information and the creation of networks of support.^19^ Participation itself may improve physical activity levels, which are known to be reduced even in people with early COPD.^20^

Isolation has a negative impact on health in general and those with a long-term condition in particular. It leads to low mood and an increasing inability to engage with self-care and self-management and has physiological effects on health.^21^ Moreover, social interaction acts positively in terms of influencing well-being and living successfully with a long-term condition.^22^ Singing may usefully contribute to reducing social isolation—a major problem in respiratory disease, particularly in those who are older and from a disadvantaged background.^23,24^

## SLH and PR

PR, a programme of supervised exercise education and support for self-management, has a grade A evidence base and should be offered to all patients who are limited by chronic respiratory disease,^25^ positioned as it is near the base of the Pyramid of Value for COPD interventions, based on its favourable cost per quality adjusted life year compared with pharmacotherapy.^26^ The concept and practice of PR and SLH overlap to an extent. Both are group activities, both include the practice and mastery of a physical activity, breathing control and relaxation and both have a holistic element.

Given PR’s powerful evidence base, it would be inappropriate to recommend SLH as an alternative to PR; however, some individuals may choose to take part in the former initially and this might make them more receptive to taking part in PR subsequently. One possibility, where SLH is available, is to hold a taster session for singing during a PR education session and participants who are interested may wish to take part in further sessions. The recent RCP PR audit has highlighted the need for signposting to activities beyond PR to help to sustain the benefits of participation, and SLH may fulfil this role. However, there is currently insufficient evidence to make any recommendation that SLH should be provided.^27^

## On what basis should SLH groups be supported?

At present, most groups are dependent on charitable support or are self-funded by participants. The British Lung Foundation currently runs a programme that involves training singing teachers and establishing classes throughout the United Kingdom. This approach has allowed an expansion of singing groups to occur, but if SLH were to be delivered sustainably at scale, health and social-care commissioners would need to support and commission ongoing provision. They will be reluctant to do this in the absence of convincing evidence for benefit, given competing demands for limited resources. For singing classes to become widely accessible to patients with lung disease, the evidence base will therefore need to be robust and be of sufficient quality for it to be included in clinical guidelines. This is not the case at present. The present *ad hoc* approach is likely to be a source of inequality as many COPD patients, a group that is socioeconomically disadvantaged and might be most likely to benefit, are less likely to have access.

## Delivery of SLH

Key to an effective singing group is a leader with the appropriate training, skills and competencies to support participants. As a recently developed therapy for respiratory disease, singing for breathing does not as yet have any accredited training programme for group leaders. Competencies, standard operating procedures or protocols will need to be developed.

In addition, singing leaders, who are not themselves health professionals, will benefit from a network of peer support and the ability on occasion to access expert advice and information about the respiratory conditions that affect the group members. Care will be needed to ensure that language is mutually understood. For example, the phrase ‘diaphragmatic breathing’ has a legitimate technical meaning in the context of singing that needs to be recognised, distinct from the scientific fact that the diaphragm is always active during inspiration.

In PR, a short, intense programme produces an improvement in exercise capacity and self-efficacy. It is hoped that this will translate into improved physical activity levels and, in particular, an ability to take part in maintenance exercises classes, not necessarily delivered by a health professional. Likewise, indefinite participation in an SLH programme, while possibly desirable for an individual, may not be sustainable. A structure where people, once they have acquired skills from a more intense time limited programme, can then take part in a less intensely supported group may be an effective approach. For some, graduating to participation in a conventional choir could be an option, though practical issues such as coughing may limit this.

## Outstanding research questions around SLH

The consensus group concluded that larger trials are needed to establish the impact of SLH on patient relevant outcomes—physical and psychological health status, exercise capacity and health resource utilisation. To date, studies have been small and short term or uncontrolled.

Further research questions include:

- Which aspects of singing training are most important for an effective programme which is attractive to patients?
- What are the objective physiological changes in people who participate in singing?
- What value-based outcome measures are required to demonstrate the impact of SLH programmes on patients’ physiological, psychological and social well-being, over a sufficient duration of follow up, necessary to persuade commissioners to provide sustainable funding for this intervention?
- What impact does SLH have on health resource utilisation?
- What is the most effective way to train and support singing group leaders?
- What is the best way to integrate singing programmes into PR and other aspects of integrated health care?
- What is the optimal duration and capacity of singing classes?

## Conclusion

There has been a rapid spread of singing groups across the United Kingdom. SLH has the potential to have a positive impact on the lives of people with lung disease, improving health status and social participation. Although early research has been encouraging, studies that are of adequate scale and duration are urgently needed to demonstrate the effectiveness of this intervention, before it can be recommended in clinical guidelines and satisfying criteria for funding by commissioners of health and social care.

## Figures and Tables

**Table 1:** Studies of Singing for Lung Health

*Study*	*Participants*	*Intervention*	*Control*	*Outcomes*
Lord *et al.*^2^	36 COPD patients, 28 completing trial (15 in singing arm). Mean age: 67.3 years. Mean FEV_1_% pred: 37.2.	30 min breathing control education provided by one senior physiotherapist followed by 6 weeks of 2×1 h group singing classes. CD to take home with homework. Royal Brompton’s ‘Help yourself—physiotherapy for people with respiratory symptoms’ booklet.	30 min breathing control education provided by a senior physiotherapist followed by standard care. Royal Brompton’s ‘Help yourself—physiotherapy for people with respiratory symptoms’ booklet.	Short Form 36 Questionnaire, Hospital Anxiety and Depression Questionnaire score, St George’s Respiratory Questionnaire, Incremental shuttle walk test, Breath hold test, single breath counting. Qualitative structured interviews.
Lord *et al.*^3^	33 COPD patients, 24 completing trial (13 in singing arm). Mean age: 68.3 years. Mean FEV_1_% pred: 53.1.	30 min breathing control education provided by one of three senior physiotherapists followed by 8 weeks of 2×1 h group singing classes led by one of the three singing teachers. Singing for breathing CD to take home with homework. Royal Brompton’s ‘Help yourself—physiotherapy for people with respiratory symptoms’ booklet.	30 min breathing control education provided by one of the three senior physiotherapists followed by standard care. Royal Brompton’s ‘Help yourself—physiotherapy for people with respiratory symptoms’ booklet. 8×1 weekly film workshops coordinated by film studies graduate.	Primary outcome measure: Short Form 36 Questionnaire score. Other outcomes: Hospital Anxiety and Depression Questionnaire score, COPD Assessment Test score, Incremental shuttle walk test, Breath hold test, single breath counting. Steps, sedentary time, physical activity duration, active energy expenditure. Qualitative structured interviews.
Bonilha *et al.*^6^	43 COPD patients, 30 completing trial (15 in singing arm). Mean age: 71.7 years. Mean FEV_1_% pred: 51.	24 weeks of ×1 weekly singing group classes led by a singing teacher and physiotherapist	24 weeks of ×1 weekly handicraft groups led by same physiotherapist as intervention group and handicraft teacher. Control group received same relaxation intervention as patients in the singing group.	Outcome measures: FVC, FEV_1_, FEV_1_/FVC, IC and ERV, maximal inspiratory and expiratory pressures, Arterial blood gases on room air, Basal dyspnoea index, Borg dyspnoea scale, SpO_2_, St George’s Respiratory Questionnaire.
Morrison *et al.*^4^	106 COPD patients, 64 patients with all postprogramme outcomes. Mean age: 69.5 years. Mean FEV_1_% pred: 54.3.	36×once weekly singing groups lasting 90 min led by a singing leaders. Group size 9–50.	No control group	Outcome measures: FEV_1_, FEV_1_%, FVC and FVC%, St George’s Respiratory Questionnaire, MRC Dyspnoea Scale, York SF-12, EuroQol 5D, Participant’s written comments after programme.
Gick and Daugherty^7^	60 Adults with self-reported asthma. Mean age 29.7 years	4×30 min sessions of group singing to karaoke backing tracks. Diaphragmatic breathing exercise session.	One group of 20 people participated in 4×30 min sessions of Karaoke only. One group of 20 people participated in diaphragmatic breathing exercise sessions only.	Outcome measures: FEV_1_, PEFR, Modified Borg Scale, Asthma Control Questionnaire, St George’s Respiratory Questionnaire, The vitality scale, General Health Questionnaire, Satisfaction with Life Scale, Positive and Negative Affect Scale, Asthma medication use, Likert scales of beliefs that singing and breathing would help asthma and the enjoyment of activities of singing and breathing, singing and breathing practice log.
Eley *et al.*^8,9^	65 Adult and child asthma patients, 38 completed study, 4 females confirmed in Eley *et al.*^9^ 3 completed the singing sessions.^8^	17–26 weekly sessions between 60–90 min within which females only were taught singing alongside storytelling, playing clapsticks, painting and boomerang throwing. Females were given mp3 player with backing tracks and voice exercises. Breathing exercises taught by professional vocal coach. Medical services staff provided information and advice about asthma management during sessions	No control group	Outcome measures: Asthma assessment questionnaire, FEV_1_, FVC, Peak flow. Comments from participants after programme.

Abbreviations: COPD, chronic obstructive pulmonary disease; FEV_1_, forced expiratory volume in 1 s; FVC, forced vital capacity; MRC, Medical Research Council; PEFR, peak expiratory flow rate.

**Table 2:** Risk of bias across studies

	*Random sequence generation*	*Allocation concealment*	*Blinding of participants and personnel*	*Blinding of outcome assessment*	*Incomplete outcome data*	*Selective reporting*	*Other bias*
Lord *et al.*^2^	Low	Low	High	Low	Low	Low	Low
Lord *et al.*^3^	Low	Low	High	Low	Low	Low	Low
Bonilha *et al.*^6^	High	High	High	Unclear	Low	Low	Low
Gick and Daugherty^7^	Low	unclear	High	High	Unclear	Low	High^a^
Morrison *et al.*^4^	High	High	High	High	Low	Low	Low
Eley *et al.*^9^	High	High	High	High	High	High	Unclear

a Asthma diagnosis not medically confirmed.

**Table 3:** Consensus statements—Singing for Lung Health

i)	Singing for Lung Health has the potential to deliver health, psychological and social benefits to people with long-term respiratory conditions.
ii)	Qualitative data from studies of Singing for Lung Health have been strongly positive.
iii)	Results of small randomised trials suggest Singing for Lung Health improves quality-of-life measures, but evidence for change in functional and health economic outcomes is so far lacking.
iv)	A distinction should be drawn between Singing for Lung Health and more generic community singing approaches, although the latter may also be valuable for participants.
v)	There is a need to define and standardise competences and training pathways for leaders of the Singing for Lung Health groups.
vi)	Appropriate outcome measures for the Singing for Lung Health groups are needed to feedback to patients and group facilitators. The simple, patient-reported outcome measure, the COPD assessment test (CAT) score, is a strong candidate measure both as feedback to patients and to ensure that the group is functioning effectively.
vii)	Robustly designed and powered trials, of adequate duration, which address patient relevant outcomes are needed before Singing for Lung Health can be adopted as an intervention to be offered routinely to people with respiratory disease.

Abbreviation: COPD, chronic obstructive pulmonary disease.

**Table 4:** Components of a Singing for Lung Health class

Physical warm up using relevant stretches and simple exercises as well as using action songs and body percussion.
Breathing exercises focussing on awareness of supporting musculature during inhalation and exhalation. Groups with a predominance of COPD patients include exercises to prolong exhalation.
Introducing unvoiced (fff, sss) and voiced (vvv, zzz) fricatives to introduce vocal fold closure and to begin to move from passive to voiced exhale.
Introducing ‘primal sounds’ (see ‘Singing and teaching singing’ by Janice L Chapman^28^) such as Hey, Ho, Ha, etc., to engage vocal mechanism and support.
Introducing a range of vocal sounds to warm up the voice, alternating different vocal qualities, range, dynamics, timbre, pitch and rhythm. These are taught in a call-and-response style to encourage an unselfconscious vocal release prior to singing songs.
Introducing a variety of more formal singing exercises to set patterns such as the first five notes of a major scale or arpeggio to start to integrate melodic patterns with the length of exhalation (e.g., the ‘Singing for Breathing’ CD, see below).
Choice from a balanced repertoire of appropriate songs that are ‘fit for purpose’ in terms of phrase lengths, breath points, lyrics, melodic challenge and range. Mixed genre, they need ideally to have a degree of difficulty with a balance between short and long phrases, range and tempo. Songs should be taught by ear in a call-and-response style, building up lyrics, melody and phrases, so all can join in. There should be a balance between sitting and standing and between using song sheets (not notation) and learning by ear.
A cool-down, guided relaxation focussing on body and breath awareness can be included as appropriate.
Practicing singing and exercises at home with a CD or tools to practice breath control such as blowing soap bubbles.
Examples of practice exercises can be found on the ‘Singing for Breathing’ CD (available from http://www.rbht.nhs.uk/about/arts/whats-on/singing-for-breathing/).

Abbreviations: CD, compact disc; COPD, chronic obstructive pulmonary disease.
