# Supplementary material for: Singing for Lung Health—a systematic review of the literature and consensus statement
Source: NPJ Prim Care Respir Med. 2016 Dec 1;26:16080–. doi: 10.1038/npjpcrm.2016.80 (PMC5131649; doi:10.1038/npjpcrm.2016.80)
Supplement: Supplementary Information [file npjpcrm201680-s1.doc]

**Singing for Lung Health – a systematic review of the literature and consensus statement.**

**ONLINE SUPPLEMENT**

^1^Adam Lewis, ^1^Phoene Cave, ^2^Myra Stern, ^3^Lindsay Welch, ^1^Karen Taylor, ^1^Juliet Russell, ^1^Anne-Marie Doyle, ^1^Anne-Marie Russell, ^4^Heather McKee, ^5^Stephen Clift, Julia Bott, ^1^Nicholas S Hopkinson.

^1^NIHR Respiratory Biomedical Research Unit at Royal Brompton and Harefield NHS Foundation Trust and Imperial College London, UK

^2^Respiratory Medicine, Whittington Health, London, UK

^3^Southampton Integrated COPD Service, Solent NHS Trust, Southampton, UK

^4^British Lung Foundation, British Lung Foundation, London, United Kingdom

^5^Sidney De Haan Research Centre for Arts and Health Canterbury Christ Church University, UK

**Corresponding author:** Dr Nicholas Hopkinson

Royal Brompton Hospital,

Fulham Rd,

London

SW3 6NP

Tel: 02073497775

Email: [n.hopkinson@ic.ac.uk](mailto:n.hopkinson@ic.ac.uk)

**Key words:** physiotherapy, COPD, art.

**Appendix A: Example of Search from Database PubMed 01/01/1980 to 14/04/2016.**

Singing AND Respiratory Disease: 228 results

Singing AND Asthma: 7 results

Singing AND COPD: 17 results

Singing AND Bronchiectasis: 1 result

Singing AND Interstitial Lung Disease: 1 result

Singing AND Obstructive Sleep Apnoea: 0 results

**Appendix B – List of studies excluded from systematic review**

Cherniack – Editorial article. Content not related to singing in respiratory disease.

Sun and Buys – Excluded due to nobody with chronic respiratory disease in the community singing groups.

Gick and Nichol – Literature review. The articles included involved singing in respiratory disease and have been included in the systematic review.

Skingley and Vella-Burrows – Review article, no studies included involved singing in chronic respiratory disease.

Canga et al – Six music therapy classes (live music visualisations, wind instrument playing and singing) during PR compared to PR alone. Pulmonary Rehabilitation lasted 24 weeks in this study.

Slikwa et al – Systematic review of music listening studies.

Irons et al original (2010) and update (2014) – systematic review – one appropriate study on singing therapy for children with cystic fibrosis. Nil appropriate adult cohort studies were included.

Pachecho et al – Ten weeks of x 1 hour singing classes in COPD patients participating in x 2 weekly 90 minutes PR maintenance groups. Singing groups led by a singing teacher and physiotherapist.

Panigrahi et al – Literature review of the role of music in the management of COPD

Herer et al – Cohort study of singing therapy during PR in 45 COPD patients. No control group was included.

Lichtenschopf et al – Four x 90 minute singing sessions during 3-4 week inpatient PR stay. Positive qualitative responses.

Grasch et al – Twice daily 10 minute singing exercises for 12 weeks in 25 COPD patients participating in PR. No group singing intervention.

Bader G et al – Conference proceedings abstract only. Non interventional study, no singing group involvement. Observational study evaluating potential effect that playing a wind instrument or singing has on reducing snoring or OSA.

Goodridge et al – 8 weeks of singing classes with 14 COPD patients in patients participating in PR compared to 5 control patients receiving usual care. No differences in outcome measures were significant between groups.

Engen - Twelve x 45 minutes of group singing classes in seven COPD patients. No control group as part of the study and some participants also participated in PR simultaneously.

**Appendix C: Results of individual studies**

| **Study/outcome** | **Lord et al (2010)** | **Lord et al (2012)** | **Bonilha et al (2009)** | **Morrison et al (2013)** | **Gick and Daugherty (2015)** | **Eley and Gorman (2010)** |
| --- | --- | --- | --- | --- | --- | --- |
| **SF36 PCS** | S: +7.5 (14.6)  C: - 3.8 (8.4)* | S: 12.9 (19.0)  C: −2.5 (11.9)* |  |  |  |  |
| **SF36 MCS** | S: + 2.5 (20.9)  C: - 3.2 (10.5) | S: 9.3 (25.3) C: 4.3 (9.0) |  |  |  |  |
| **SF12 PCS** |  |  |  | S: 0.09 (2.47) |  |  |
| **SF12 MCS** |  |  |  | S: 1.50 (4.81) |  |  |
| **HAD (A)** | S: - 1.1 (2.7) C: + 0.8 (1.7)* | S: −0.8 (3.6) C: −0.9 (2.3) |  |  |  |  |
| **HAD (D)** | S:- 1.1 (2.5) C: - 0.1 (1.7) | S:−1.3 (3.8) C: −0.7 (1.6) |  |  |  |  |
| **SGRQ** | S: - 1.1 (10.6)  C: - 0.4 (5.6) |  | S:–5.9 (5.8)  C: –5.0 (7.8) | S: -3.29 (5.69)* | S: 0.08  B: -0.46  S + B: 0.51 |  |
| **ISWT (M)** | S:+ 26 (52.6)  C: + 11.3 (83.0) | S:−7.2 (46.1)  C: 14.5 (38.0) |  |  |  |  |
| **Breath Hold Test** | S: - 0.3 (6.9) C: + 5.3 (5.7)* | S: −1.64 (4.1)  C: 2.39 (7.8) |  |  |  |  |
| **Single Breath Counting** | S: + 0.3 (7.7)  C: + 2.0 (2.7) | S: 1.5 (7.1) C: 7.0 (7.8) |  |  |  |  |
| **SPO_2_ recovery** | S: + 47.3 (67.6)  C: + 32.2 (124.7) |  |  |  |  |  |
| **HR recovery** | S: + 29 (63.8)  C:+ 19.4 (110.0) |  |  |  |  |  |
| **CAT** |  | S:−1.1 (8.3) C: 0.7 (5.6) |  |  |  |  |
| **MRC dyspnoea** |  |  |  | S: -0.13 (0.42) |  |  |
| **Steps** |  | S: −763 (1647)  C: 1011 (1003) |  |  |  |  |
| **Sedentary Time** |  | S: −35.9 (127.3)  C: −27.3 (67.0) |  |  |  |  |
| **PA duration** |  | S: −92.7 (216.9)  C: 49.5 (40.9) |  |  |  |  |
| **AEE** |  | S:−144.2 (436.0) C: 228.8 (146.3) |  |  |  |  |
| **FVC** |  |  | S: –0.14 (0.48)  C: –0.10 (0.30) | S: 0.11 ( 0.19)* |  |  |
| **FVC % Pred** |  |  |  | S: 3.63 ( 6.70)* |  |  |
| **FEV_1_** |  |  | S: –0.03 (0.31)  C: 0 (0.14) | S: 0.03 ( 0.59)* | S: 0.07  B: -0.09  S+B: 0.05 |  |
| **FEV_1_% Pred** |  |  |  | S: 1.94 (2.72) * |  |  |
| **FEV_1_/FVC** |  |  | S: 1.9 (8.3) C: 1.5 (2.9) |  |  |  |
| **IC (change 2 minutes post intervention)** |  |  | S: –0.09 (0.3)  C: 0.07 (0.3)* |  |  |  |
| **ERV** |  |  | S: 0.06 (0.4) B: –0.11 (0.2) |  |  |  |
| **MIP** |  |  | S: 3.0 (19.2)  C: –1.0 (15.5) |  |  |  |
| **MEP** |  |  | S: 3.0 (17.2)  C: -11.3 (20.2)* |  |  |  |
| **ABG A-a O_2_** |  |  | S: –0.4 (0.8) C: 0.1 (1.6) |  |  |  |
| **BDI** |  |  | S: 0.7 (1.2)  C: 0.3(1.7) |  |  |  |
| **BORG change through intervention** |  |  |  |  | S: -0.36  B: - 0.09  S + B: -0.43 |  |
| **BORG (change 2 mins post intervention)** |  |  | S: 0.5 (0.7)  C: –0.3 (0.6)* |  |  |  |
| **SpO_2_ (during intervention)** |  |  | S:1.6(1.8)* C: 0(1.2) |  |  |  |
| **EuroQOL – 5D utility** |  |  |  | S: 0.04  (0.09) |  |  |
| **EuroQOL – 5D VAS** |  |  |  | S: 3.24  (8.88) |  |  |
| **PEFR** |  |  |  |  | S: 69.9  B: 27.6  S + B: 43.14 |  |
| **PEF %** |  |  |  |  |  | S: 27.6 |
| **ACQ** |  |  |  |  | S: -0.01  B: - 0.23  S+B: -0.11 |  |
| **The Vitality Score** |  |  |  |  | S: 0.89  B: 0.16  S+B: 0.47 |  |
| **GHQ** |  |  |  |  | S: -4.47  B: -1.00  S + B: -3.81 |  |
| **SWLS** |  |  |  |  | S: 1.64  B: 1.3  S+B: 0.73 |  |
| **PANAS PA** |  |  |  |  | S: 2.12  B: 2.4  B + S: - 0.34 |  |
| **PANAS NA** |  |  |  |  | S: -1.65  B: -2.33  B+S: -3.94 |  |

S = singing group C = control group B = Breathing only group B + S: Breathing and singing. SF36 PCS = Short Form 36 Physical Component Score. SF36 MCS = Short Form 36 Mental Component Score SF12 PCS = Short form 12 physical component score SF12 MCS = Short form 12 Mental Component Score. HAD (A) = Hospital Anxiety and Depression anxiety score. HAD (D) = Hospital Anxiety and Depression depression score. SGRQ = St Georges Respiratory Questionnaire. ISWT (M) = Incremental Shuttle Walk Test (Metres). SpO2 recovery = Time in seconds for blood oxygen saturation to recovery to baseline after completing the ISWT. HR recovery = Time in seconds for heart rate to recover to baseline after completing the ISWT. CAT = Chronic Obstructive Pulmonary Disease Assessment Test score. MRC dyspnoea = Medical Research Council dyspnoea scale. PA duration = Time in minutes spent in moderate intensity physical activity. AEE = Active Energy Expenditure in Kilojoules. FVC = Forced Vital Capacity in litres. FVC % Pred = The predicted Forced Vital Capacity according to age, sex and height of participant FEV_1_ = Forced Expiratory Volume in the first second of expiration from maximal inspiration. FEV_1_% = The predicted Forced Expiratory Volume in the first second of expiration from maximal inspiration. FEV_1_/FVC = The ratio of Forced Expiratory Volume in the first second of expiration from maximal inspiration over the Forced Vital Capacity. IC = Inspiratory Capacity. ERV = Expiratory Reserve Volume. MIP = Maximal expiratory pressure. MEP = Maximal expiratory pressure Volume. ABG A-a O2 = Arterial Blood Gas result of Arterial-alveolar gradient oxygenation. BDI = Basal Dyspnoea Index. BORG = BORG dyspnoea scale. SpO2 = oxygen saturation. EuroQOL – 5D utility = European Quality of Life 5 dimensions utility score. EuroQOL – 5D VAS = European Quality of Life 5 dimensions Visual Analogue Scale. PEFR = Peak expiratory Flow Rate. PEF% = Percent of predicted Peak Expiratory Flow for participant age height and sex. ACQ = Asthma Control Questionnaire. GHQ = General Health Questionnaire. SWLS = Satisfaction with Life Scale. PANAS PA = Positive Affect And Negative Affect Scale Positive Affect score. PANAS NA = Positive Affect And Negative Affect Scale Negative Affect score. All results are presented as a change pre-post intervention and standard deviations are bracketed. If results are significant they are given an asterix. If Pre-Post intervention standard deviations were not presented or raw data from each outcome measure were not available from the original article these have been not been included in the table. In Gick and Daugherty (2013) The data sets used are Pre intervention (week 1) and post intervention (week 4).

**References**

1. Cherniack NS. Singing the Chronic Obstructive Pulmonary Disease Blues. *Respiration* 2002: 69(2): 115-116.

2. Sun J, Buys N. Participatory community singing program to enhance quality of life and social and emotional well-being in Aboriginal and Torres Strait Islander Australians with chronic diseases. *International Journal on Disability and Human Development* 2013: 12(3): 317-323.

3. Gick ML, Nicol JJ. Singing for respiratory health: theory, evidence and challenges. *Health promotion international* 2015.

4. Skingley A, Vella-Burrows T. Therapeutic effects of music and singing for older people. *Nurs Stand* 2010: 24(19): 35-41.

5. Canga B, Azoulay R, Raskin J, Loewy J. AIR: Advances in Respiration - Music therapy in the treatment of chronic pulmonary disease. *Respir Med* 2015: 109(12): 1532-1539.

6. Sliwka A, Wloch T, Tynor D, Nowobilski R. Do asthmatics benefit from music therapy? A systematic review. *Complement Ther Med* 2014: 22(4): 756-766.

7. Irons JY, Kenny DT, Chang AB. Singing for children and adults with bronchiectasis. *The Cochrane database of systematic reviews* 2010(2): Cd007729.

8. Irons JY, Petocz P, Kenny DT, Chang AB. Singing as an adjunct therapy for children and adults with cystic fibrosis. *The Cochrane database of systematic reviews* 2014: 6: Cd008036.

9. Pacheco C, Costa A, Amado J, Almeida P. Singing in chronic obstructive pulmonary disease patients: A pilot study in Portugal. *Revista portuguesa de pneumologia* 2014: 20(4): 225-228.

10. Panigrahi A, Sohani S, Amadi C, Joshi A. Role of music in the management of chronic obstructive pulmonary disease (COPD): a literature review. *Technology and health care : official journal of the European Society for Engineering and Medicine* 2014: 22(1): 53-61.

11. Herer B. [Outcomes of a pulmonary rehabilitation program including singing training]. *Revue des maladies respiratoires* 2013: 30(3): 194-202.

12. Lichtenschopf A, Skarek E, Müller R. Singen als Erweiterung des therapeutischen Spektrums bei der Behandlung der COPD. *Atemwegs und Lungenkrankheiten* 2014: 40(6): 261-264.

13. Grasch A, Boley TM, Colle J, Henkle JQ, Todd SO, Hazelrigg SO, eds. Daily Singing Practice as a Means of Improving Pulmonary Function and Quality of Life in Emphysema Patients. 03/07/2013 ed, 2013.

14. Bader G, Bodden A, Ullrich A. Does singing or playing a wind instrument have any impact on snoring or OSA? In: Journal of Sleep Research Conference: 20th Congress of the European Sleep Research 2010; Lisbon, Portugal.: Blackwell Publishing Ltd; 2010.

15. Goodridge D, Nicol JJ, Horvey KJ, Butcher S. Therapeutic Singing as an Adjunct for Pulmonary Rehabilitation Participants With COPD: Outcomes of a Feasibility Study. *Music and Medicine* 2013: 5(3): 169-176.

16. Engen RL. The singer's breath: implications for treatment of persons with emphysema. *J Music Ther* 2005: 42(1): 20-48.

17. Lord VM, Cave P, Hume VJ, Flude EJ, Evans A, Kelly JL, Polkey MI, Hopkinson NS. Singing teaching as a therapy for chronic respiratory disease--a randomised controlled trial and qualitative evaluation. *BMC Pulm Med* 2010: 10: 41.

18. Lord VM, Hume VJ, Kelly JL, Cave P, Silver J, Waldman M, White C, Smith C, Tanner R, Sanchez M, Man WD, Polkey MI, Hopkinson NS. Singing classes for chronic obstructive pulmonary disease: a randomized controlled trial. *BMC Pulm Med* 2012: 12(1): 69.

19. Bonilha AG, Onofre F, Vieira ML, Prado MY, Martinez JA. Effects of singing classes on pulmonary function and quality of life of COPD patients. *International journal of chronic obstructive pulmonary disease* 2009: 4(1): 1-8.

20. Morrison I, Clift S. A UK feasibility study on the value of singing for people with Chronic Obstructive Pulmonary Disease (COPD). *UNESCO Observatory Multi-Disciplinary Journal in the Arts* 2013: 3(3): 1-19.

21. Gick ML, Daugherty C. Changes in Spirometry, Quality of Life and Well-Being in Persons with Asthma following Singing, Diaphragmatic Breathing, and Singing and Diaphragmatic Breathing: A Pilot Study. *Music and Medicine* 2015: 7(4): 40-49.

22. Eley R, Gorman D. Didgeridoo playing and singing to support asthma management in Aboriginal Australians. *J Rural Health* 2010: 26(1): 100-104.
